# Maternal *Lactobacillus reuteri* supplementation shifts the intestinal microbiome in mice and provides protection from experimental colitis in female offspring

**DOI:** 10.1096/fba.2021-00078

**Published:** 2021-11-01

**Authors:** Mahesh Krishna, Melinda Engevik, Karen Queliza, Savini Britto, Rajesh Shah, Wenly Ruan, Hongtao Wang, James Versalovic, Richard Kellermayer

**Affiliations:** ^1^ Johns Hopkins School of Medicine Baltimore Maryland USA; ^2^ Section of Pediatric Gastroenterology Baylor College of Medicine Houston Texas USA; ^3^ Department of Pathology & Immunology Baylor College of Medicine Houston Texas USA; ^4^ Pediatric Gastroenterology, Hepatology and Nutrition Memorial Sloan Kettering Cancer Center New York New York USA; ^5^ Department of Medicine Baylor Scott and White Austin Texas USA; ^6^ USDA/ARS Children's Nutrition Research Center Texas Children's Hospital Houston Texas USA

**Keywords:** inflammatory bowel disease, *Lactobacillus*, microbiota, pediatric, probiotic, stochastic

## Abstract

The purpose of our experiment was to explore how stochastic (inter‐individual variation) gut microbiome composition may link to inflammatory bowel disease (IBD) susceptibility and guide the development of a perinatal preventative probiotic. Dextran sodium sulfate (DSS) was introduced to C57BL/BJ mice to induce acute colitis as a model of IBD. Potentially protective bacteria were identified using a discovery‐validation cohort approach toward stochastic DSS susceptibility. *Lactobacilli* (two different cocktails of *L*. *reuteri* and *L*. *johnsonii* strains) or control media were supplemented by mouth to dams prior to delivery and during lactation (i.e., perinatal probiotic). The pups were evaluated for DSS susceptibility at young adulthood. Fecal *Lactobacillus* was increased in the DSS‐resistant mice in both the discovery and validation cohorts. Maternal supplementation of female offspring with an *L*. *reuteri* cocktail (strains 6798‐1, 6798‐jm, and 6798‐cm) induced progressive microbiome separation and protection against colitis by young adulthood. Maternal supplementation of *L*. *reuteri* could confer protection against DSS colitis in young adult female mice. This work is the first to exploit stochastic mammalian microbiome variation to guide microbial therapeutic identification. Our findings underscore neonatal microbiome plasticity and set the stage for the potential development of perinatally deliverable protective probiotics against human IBD.

**Abbreviations**
CD: Crohn's disease
DSS: dextran sulfate sodium
DSS‐R: DSS‐resistant
DSS‐S: DSS‐sensitive
IBDs: inflammatory bowel diseases
LJ‐PS: *L*. *johnsonii* probiotic supplementation
LR‐PS: *Lactobacillus reuteri* probiotic supplementation
PCoA: principal coordinate analysis
PX: postnatal day X
UC: ulcerative colitis

## 1 INTRODUCTION

Inflammatory bowel diseases (IBDs) are categorized into two major subtypes: ulcerative colitis (UC) and Crohn's disease (CD). These disorders are chronic inflammatory conditions of the digestive tract with unknown etiology. The peak onset of IBD occurs during young adulthood (second or third decade of life).^1^ IBD incidence has increased in developed countries over the second half of the 20th century and more recently in populations adopting a westernized lifestyle.^2^, ^3^ This increasing incidence, along with the difficulty to treat the diseases has led to a large healthcare and economic burden worldwide, including more than $5.5 billion annual cost in the USA alone.^4^ Therefore, novel preventative and therapeutic measures that could reduce the morbidity and economic burden of these diseases are sorely needed.

Genetic predisposition, environmental influences, the immune system, and the gut microbiome are essential components in the development of IBD.^5^ An altered or dysbiotic gut microbial system has been implicated in the pathogenesis of IBD.^6^, ^7^ Monozygotic twin discordance in IBD is as high as 60% in CD and 85% in UC (reviewed in Ref. [4]). Monozygotic twins (genetically identical) are most commonly exposed to the same environment, and have similar microbiomes^8^; theoretically a concordance rate close to 100% should be observed.^9^, ^10^ Since this is not the case, stochastic variation in individual biological systems must play an important role in the etiology of IBD.^6^, ^11^, ^12^ Therefore, the gut microbiome is a highly plausible target where stochastic, environmentally responsive changes may induce susceptibility toward IBD, and stochastic interactions may even be at play after the onset of the disease. Intriguingly, one of the largest undertakings on the human gut microbiome to date has recently concluded that dysbiotic periods in IBD patients are “potentially stochastic”.^13^ Even more recently, Clooney et al.^14^ recognized that 90.3% of microbiome compositional variance is stochastic or unexplained in 531 IBD patients and 161 controls from Ireland and Canada.

In humans, early microbiome development has been found to impact disease susceptibility.^15^ Studies by Yatsunenko et al.^16^ and Zhou et al.^10^ found that inter‐individual variation was greater in younger children compared to older individuals. Yatsunenko et al.^16^ observed greater microbiome variation in children younger than 3 years of age, compared to adults in Malawi, Venezuela, and the United States, suggesting that this observation is independent of geography. Zhou et al.^10^ found the gut microbiome to stabilize after 1 year of age, and highlighted a change in microbial metabolic genes when comparing infants younger than 1 with infants older than 1. These observations suggest an age‐related metabolic link that influences the variation in microbiome diversity. Therefore, it may be within the first few years of life that the microbiome undergoes stochastic selectivity. This random selection is influenced by genetic^17^ and epigenetic^18^ predisposition, as well as postnatal environmental influences (such as early antibiotic exposure^19^), which can lead to a microbiome‐gut interactome^5^ that is more, or less susceptible toward developing IBD later in life.^20^

In this study, we aimed to examine how stochastic microbiome composition may link to IBD susceptibility. We did this to determine if such compositional associations may provide a means to modulate early microbiome development in order to decrease subsequent susceptibility to intestinal inflammation. Inbred mice, although imperfect models of IBD,^21^ are uniquely advantageous to examine stochastic microbiome variation, but they are less commonly recognized for this purpose. In particular, due to their genetically identical nature and their identical nurture within research facilities, they are optimal for studying random variations within the microbiome, largely independently from genetics and environment. In mice, inter‐individual variation, or stochasticity, has been found to have a significant effect on the microbiome.^22^ In this particular study by Hildebrand, et al.,^22^ inter‐individual variation was found to contribute to 45.5% of the variance in the gut microbiome. Previous research indicated that inter‐individual microbiome variation is greater in some mouse strains like C57BL6/J compared to others such as C3HRI, DBAJR, and WSB.^23^, ^24^ In a study by McCafferty et al.,^25^ stochastic changes within each cage of mice were found to be the main cause of cage effects. Hufeldt et al.^26^ observed that inter‐individual variation in the gut microbiome was decreased when the female breeders were closely related. Another study found that wild type (WT) C57BL/6 mice from vendors were more susceptible to developing colitis than WT C57BL/6 animals maintained and bred within a smaller animal facility.^27^ Even though random inter‐individual variation has been observed in the mammalian microbiome, this variation has surprisingly not been studied in the context of intestinal inflammation, specifically in respect to IBD.

Here, we examined how random microbiome variation at different stages of development associates with early adulthood (when the incidence of IBD peaks in humans^1^) susceptibility to dextran sulfate sodium (DSS) induced colitis. Our primary outcome was weight loss as an inherent surrogate marker of colitis severity in this model. Indeed, we have recently underscored that weight loss is a sufficient and economically feasible single outcome measure of murine DSS colitis.^28^ Our goal was to identify potential probiotics that, if given during early postnatal development (even in a transient fashion) may prevent/decrease microbiome evolution toward an IBD prone composition.

## 2 MATERIALS AND METHODS

### 2.1 Animals in discovery‐validation cohort

Our novel approach is outlined in Figure S1. A discovery‐validation cohort method was used to investigate the impact of stochastic microbiome variation on dextran sodium sulfate (DSS) susceptibility. A discovery cohort of newly purchased 8‐week‐old C57BL/6J mice (*n* = 30) was studied after cage mixing and acclimation to our vivarium (i.e., vendor‐based discovery experiment). Stool in the discovery cohort was collected before experimental colitis induction at postnatal day 70 (P70, i.e., young adult mice). For the validation cohort, three breeding trios (one male, two females) of C57BL/6J mice were set up in our vivarium, and pups (*n* = 31) were studied (i.e., vivarium‐based validation experiment). Stool in the validation cohort was collected at P21 (weaning) and prior to experimental colitis induction at P70. For both cohorts, only male mice were used to limit potential gender‐based microbial and DSS sensitivity variation.^27^

### 2.2 Administration of dextran sodium sulfate

At P70, 2.5% (wt/vol) dextran sodium sulfate (DSS) (MW = 36,000–50,000, MP Biomedicals, LLC) was administered to the mice through drinking water. Weight was monitored daily and DSS was stopped once mice started losing 5% of their original body weight.

### 2.3 *16S rRNA* sequencing

The fecal microbiomes were analyzed by high throughput sequencing of the bacterial *16S rRNA* gene at the Alkek Center for Metagenomics and Microbiome Research, and data was analyzed through ATIMA (Agile Toolkit for Incisive Microbial Analyses) developed by the Center for Metagenomics and Microbiome Research at Baylor College of Medicine, identical to Ihekweazu, et al.^29^

### 2.4 Lactobacillus culture

The second portion of the study, as a proof of concept, examined the impact of early life *Lactobacillus* supplementation on subsequent DSS susceptibility in young adulthood (Figure S2). We selected *L*. *reuteri* and *L*. *johnsonii* species because they have been previously found to promote gut barrier function.^30^ *L*. *reuteri* strains 6798‐1, 6798‐jm, and 6798‐cm and *L*. *johnsonii* strains 4901, 4903, 4931 were previously identified from mouse feces.^31^ Only these two *Lactobacillus* species were used due to available expertise with the specific species at Baylor College of Medicine and previous postnatal studies in humans indicating early postnatal effects from *Lactobacilli* (see Section 4). Both cocktails were created from *Lactobacillus* strains originating from mice without colitis (Swiss Webster and inducible nitric oxide synthetase‐deficient C57BL/6 mice).^31^ These *Lactobacilli* were cultured in an anaerobic workstation (Anaerobe Systems AS‐580) in a mixture of 5% CO_2_, 5% H_2_, and 90% N_2_. Colonies were grown in de Man, Rogosa and Sharpe (MRS) agar (Difco) anaerobically at 37°C overnight. Single colonies were used to inoculate MRS medium and grown anaerobically at 37°C overnight. After growth, *Lactobacilli* were pelleted by centrifuging at 5000 *g* for 5 min. Bacterial cells were washed three times with sterile anaerobic PBS to wash away residual MRS, and the bacterial pellet was suspended in anaerobic phosphate‐buffered saline (PBS) at 10^9^ colony forming units (CFUs) per ml.

### 2.5 Lactobacillus supplementation

To determine if transient maternal supplement of *Lactobacilli* transfers a protective microbiome, we orally gavaged C57BL/6 dams (*n* = 6, 2 per group) with phosphate‐buffered saline (PBS), *L*. *reuteri* cocktail (strains 6798‐1, 6798‐jm, and 6798‐cm) or *L*. *johnsonii* cocktail (strains 4901, 4903, 4931). Both male and female offspring were subsequently examined. There were 39 offspring mice given the *L*. *reuteri* cocktail (24 male, 15 female), 41 given the *L*. *johnsonii* cocktail (19 male, 22 female), and 36 given PBS as a control (20 male, 16 female). The *L*. *reuteri* (strains 6798‐1, 6798‐jm, and 6798‐cm) and *L*. *johnsonii* (strains 4901, 4903, 4931)^31^ cocktails were administered to the dams 1 week prior to giving birth, every other day, based on a previous experiment in which effective colonization of *Bifidobacterium* was transferred to the pups by gavaging the dams.^32^ After birth, dams were left alone for a week before restarting every other day oral gavage for a week until weaning. The goal was to supplement the pups the desired *Lactobacillus* strains 1 week prior to delivery, and as intensely (but practically and safely) as possible through the last 2 weeks of lactation, through maternal transmission. Pre‐ and postnatally (during last 2 weeks of lactation) supplemented pups were weaned to a standard diet of our vivarium (PicoLab Select Rodent 50 IF/6F) and were maintained in 4/cage setting, with gender and with infantile probiotic treatment‐based separation. DSS (2.5%) was started on P70 and continued for 6 days. Daily weights were collected. Weight loss was examined as the single, sufficient and economically feasible measure of DSS colitis severity, as underscored by us recently.^28^ Pup stools were collected at various points (P21 (weaning), P30, and P70 (pre‐DSS)). Fecal pellets from a select number of female mice (since only those had a variation in colitis phenotype) at each time point were sent for analysis to the Alkek Center for Metagenomics and Microbiome Research at Baylor College of Medicine.

### 2.6 Statistical analysis

For analysis of genera separation, the Mann–Whitney *U*‐test was utilized for comparisons between two groups (denoted as direct comparison), while the Kruskal–Wallis test was used for comparisons of three or more groups. The level of significance was set at *p* < 0.05. The protocol was approved by the Institutional Animal Care and Use Committee of Baylor College of Medicine (AN‐5351).

Analysis of variance (ANOVA) with repeated measures was performed using SAS 9.4 (SAS Institute Inc., 2007) to assess the percent changes in body weight in response to DSS‐induced colitis in P70 female pups that were exposed to maternal supplementation with *L*.*reuteri* (*n* = 15) versus control media (*n* = 16). The ANOVA with repeated measures was performed using PROC MIXED to assess the percent changes in body weight with main effects for treatment (*L. reuteri* or control media) and days post‐DSS treatment (days 0–11) and the treatment by day interaction. The Fisher's Least Significance Difference procedure was used to assess the nature of the differences observed with the significant treatment by day interaction. Statistical significance was set at *p* < 0.05.

We identified transient weight loss in a subgroup of mice on the second day of the experiment due to accidental water loss and transient dehydration. In order to address if this incident potentially influenced the experimental outcome, post‐hoc analysis was conducted with the exclusion of mice that were affected (Figure S3). The results of the analysis were similar to the original observations (see Figure 3 later), indicating that the transient water loss did not affect the difference we observed between the *L*. *reuteri* supplemented and the control females.

## 3 RESULTS

### 3.1 Discovery cohort

**FIGURE 1:**
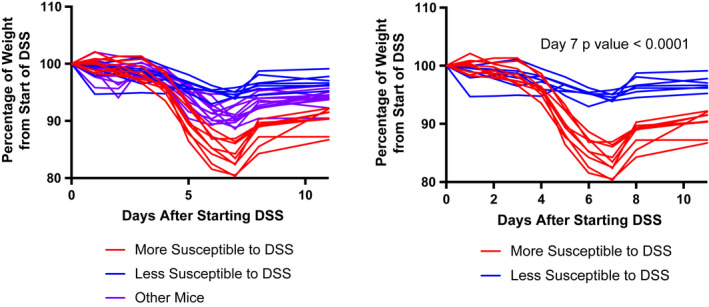
Discovery cohort weights. Weight graphs of all mice in discovery cohort (left graph) and comparison of mice we arbitrarily identified as most (*n* = 9)/least (*n* = 7) susceptible to DSS after experiment (right graph). The *p* value between these two groups at day 7 was <0.0001 [Colour figure can be viewed at wileyonlinelibrary.com]

**FIGURE 2:**
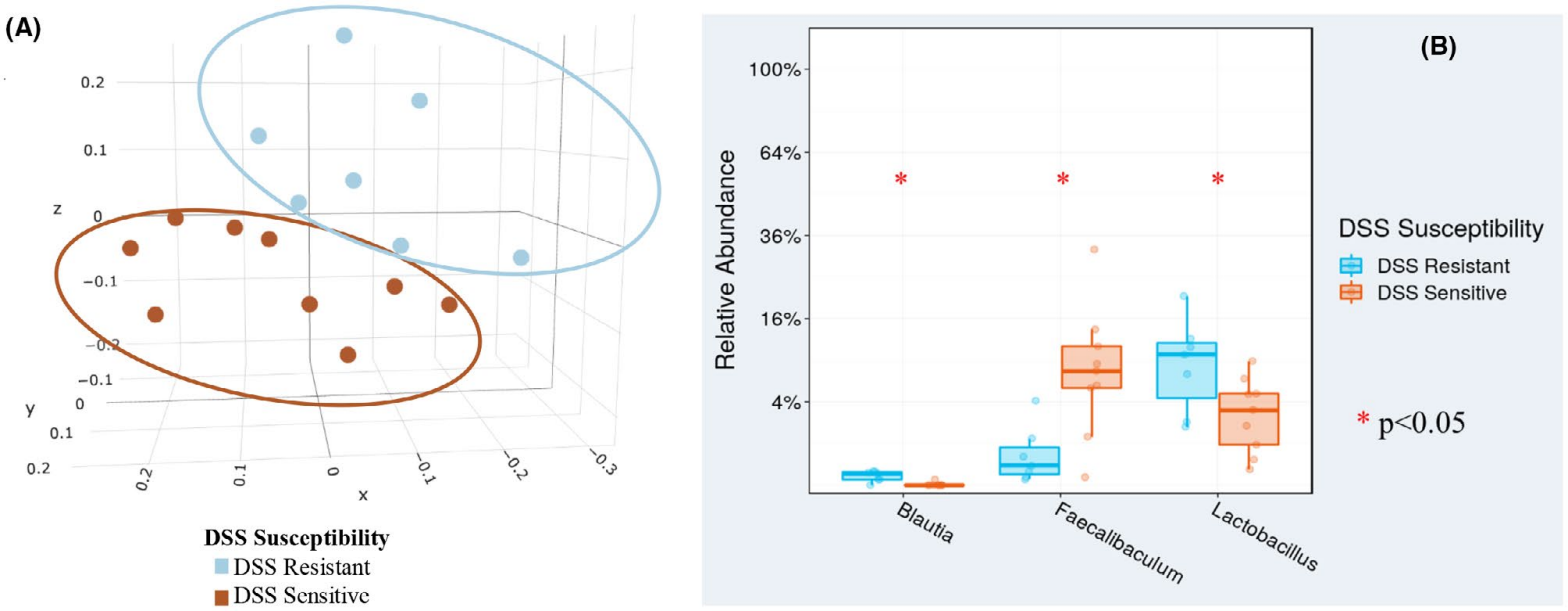
Principal coordinate analysis (PCoA) indicated microbiome separation and increased abundance of *Lactobacillus* pre‐DSS. (A) Weighted PCoA indicated that there was a distinct microbial separation (*p* = 0.001) between the mice that lost the most weight (DSS‐sensitive: DSS‐S) and the mice that lost the least weight (DSS‐resistant: DSS‐R) after start of DSS. (B) *Blautia*, *Faecalibaculum*, and *Lactobacillus* separated most at pre‐DSS between the DSS resistant and DSS sensitive microbiomes (*Blautia*: *p* = 0.003; *Faecalibaculum*: *p* = 0.01; *Lactobacillus*: DSS‐S mean = 3.53%, DSS‐R mean = 9.32%, *p* = 0.04) [Colour figure can be viewed at wileyonlinelibrary.com]

In the initial discovery cohort, we created arbitrary groups of DSS‐resistant (DSS‐R) and DSS‐sensitive (DSS‐S) animals based on separation by weight loss during the experimentally induced colitis (Figure 1). These arbitrary groups were designated after the experiment, based on the theoretical approach described in Figure S1B. When comparing the microbiomes between the DSS‐S and DSS‐R mice at P70 (pre‐DSS), a distinct separation was found by Principal Coordinate Analysis (PCoA) (Figure 2A; *p* = 0.001). Three genera were found to significantly differ in abundance between the DSS‐R and the DSS‐S classified microbiomes (Figure 2B). *Lactobacillus* and *Blautia* were increased in DSS‐R mice, while *Faecalibaculum* was increased in DSS‐S animals. Therefore, *Lactobacillus* and *Blautia* were associated with protection against colitis in the discovery cohort. A validation cohort observing the DSS‐R and DSS‐S microbiomes at P21 (and other time points) was conducted to search for a developmental probiotic candidate.

### 3.2 Validation cohort

**FIGURE 3:**
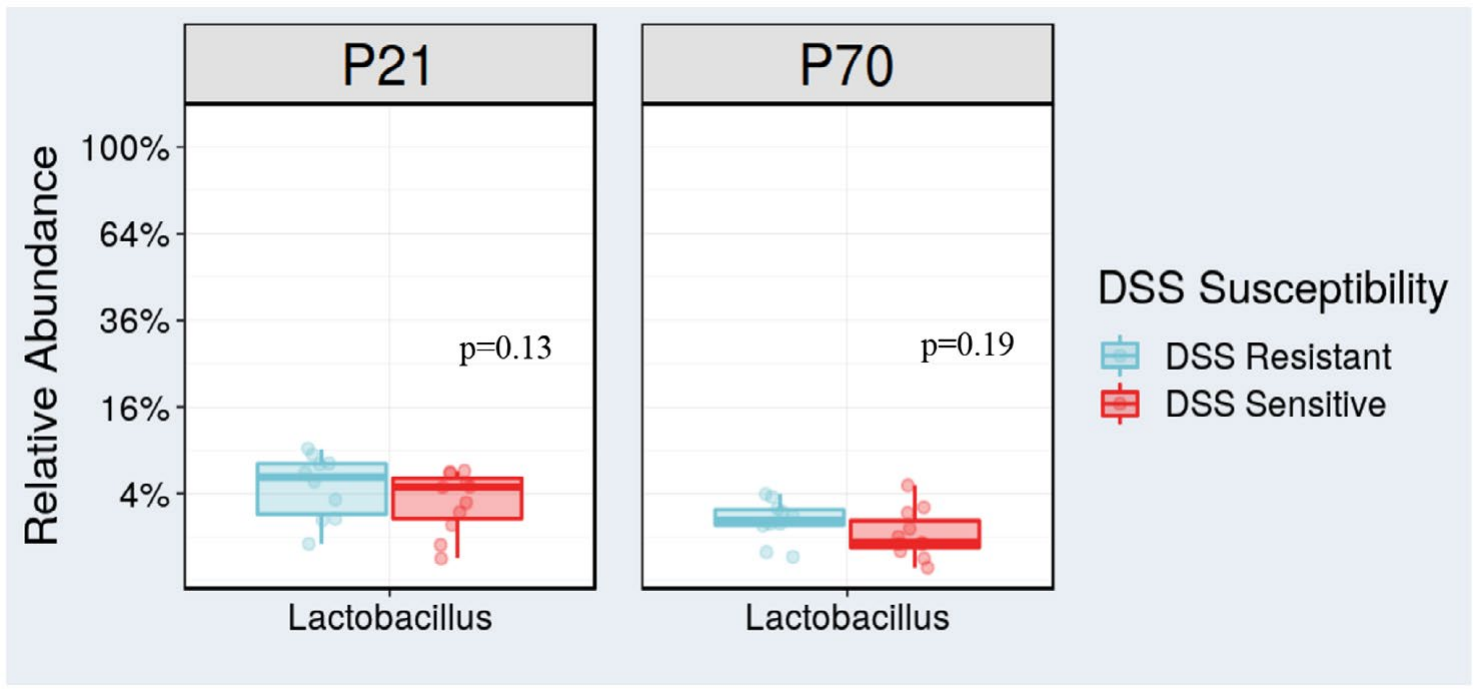
The validation cohort supported that increased *Lactobacillus* pre‐DSS may protect against colitis. *Lactobacillus* was increased in the DSS‐R group (mean = 2.07%) versus the DSS‐S group (mean = 1.40%) at P70. Although the difference in *Lactobacillus* was not significant (*p* = 0.19), it was one of the distinguishing genera between DSS‐R and DSS‐S in the validation cohort, and the results were consistent with the discovery cohort observations. Additionally, at P21, the DSS‐R group (mean = 5.28%) had higher abundance of *Lactobacillus* compared to DSS‐S (mean = 3.8%), which also did not reach statistical significance but supported the consistent trend (*p* = 0.13) [Colour figure can be viewed at wileyonlinelibrary.com]

In the validation cohort, DSS sensitivity‐based separation was not as distinct as in the discovery cohort (Figure S4). Correspondingly, beta diversity was not significantly different between DSS‐R and DSS‐S microbiomes (not shown). *Lactobacillus* was the only genus from the discovery and validation cohorts that was consistently associated with protection against colitis, although this separation between DSS‐R and DSS‐S groups was not significant either at P21 or P70 in the validation cohort (Figure 3). Nevertheless, the novelty of our approach and the consistent findings (despite the relatively small number of mice studied) with respect to *Lactobacilli* between our discovery and validation experiments, provided enough evidence for us to pursue this genus as a potential developmental probiotic in a proof‐of‐concept manner.

### 3.3 *Lactobacillus* supplementation

**FIGURE 4:**
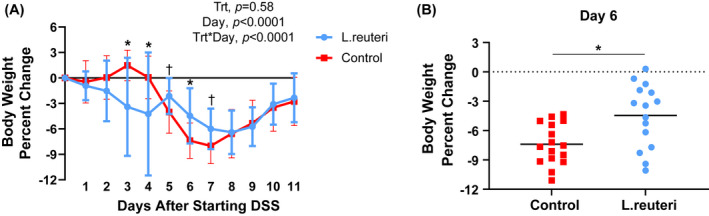
Female offspring may be protected by maternal *L*. *reuteri* supplementation. Statistical analysis included a two‐way ANOVA with repeated measures. (A) The P70 female pups supplemented with *L*. *reuteri* (*n* = 15) lost significantly less weight (were more DSS resistant) at day 6 compared to the female pups just given control media (*n* = 16) after the start of DSS. Values are means ± SD. (B) Individual data representation of P70 female pups supplemented with *L*. *reuteri* (*n* = 15), which lost significantly less weight (were more DSS resistant) at day 6 than control. Bars represent the means. *p < 0.05, ^†^ *p* < 0.10 [Colour figure can be viewed at wileyonlinelibrary.com]

**FIGURE 5:**
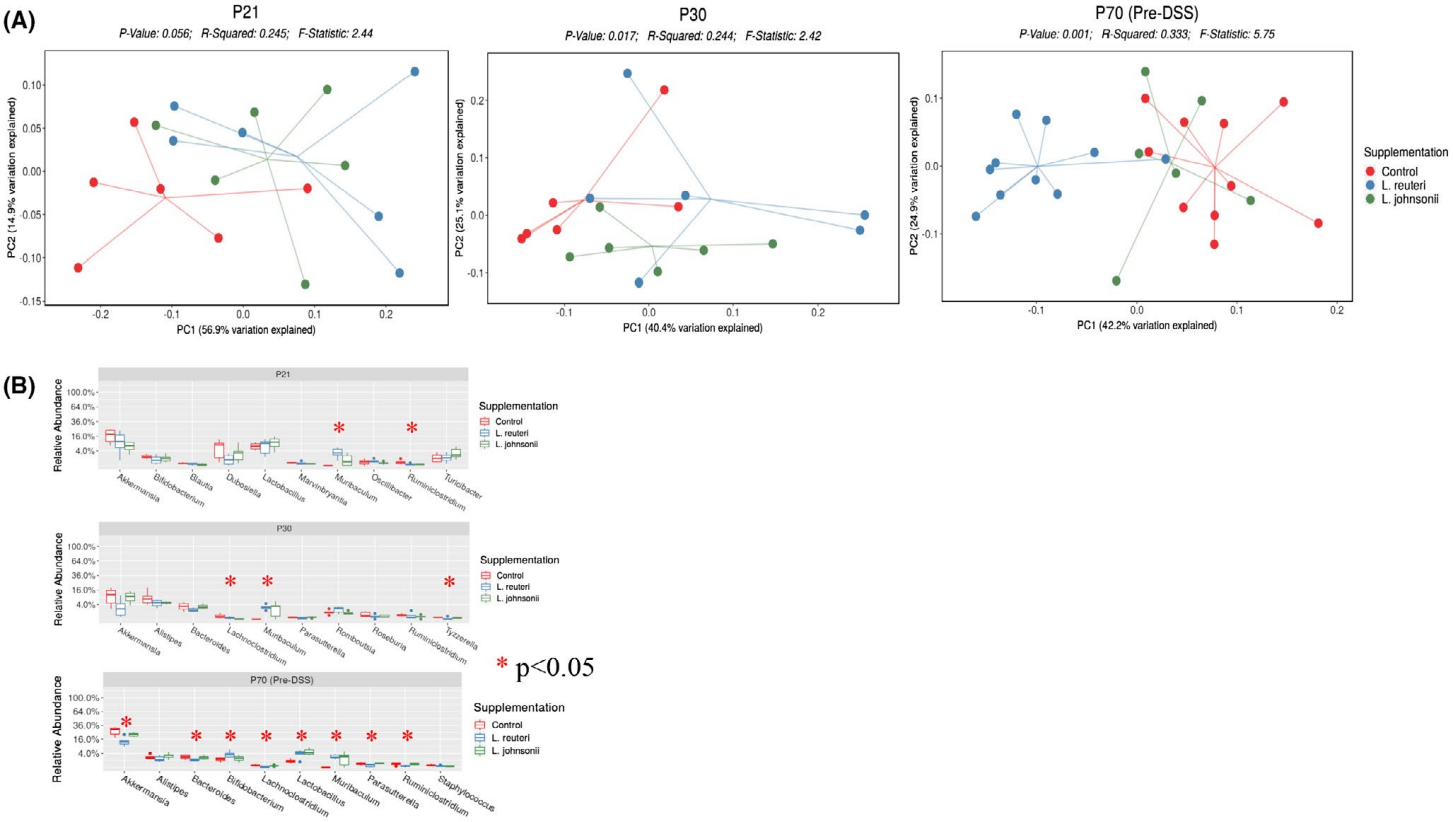
Differences in female gut microbiome based on supplementation. (A) Principal coordinate analysis (PCoA) of female pups’ microbiome samples was done at different time points: P21, P30, and P70. At P21, the control group microbiomes separated from *Lactobacillus*‐supplemented groups (*p* = 0.056). *L*. *reuteri*, *L*. *johnsonii*, and control groups progressively separated with aging (P30; *p* = 0.017 and P70; *p* = 0.001). By P70, *L*. *reuteri* supplemented female pups separated from *L*. *johnsonii* and control groups. (B) Comparisons of the female pups’ microbiomes were done at the genus level. The top 10 genera of microbial differences between *L*. *reuteri*, *L*. *johnsonii*, and control groups are shown at each time point. For P21, *Muribaculum* (*p* = 0.0038) and *Ruminiclostridium* (*p* = 0.0314) were the only genera with significant separation as *p* < 0.05. For P30, *Lachnoclostridium* (*p* = 0.0087), *Muribaculum* (*p* = 0.0131), and *Tyzzerella* (*p* = 0.0491) were significant. Finally, at P70, eight out of the top 10 genera separated significantly [Colour figure can be viewed at wileyonlinelibrary.com]

*Lactobacillus reuteri* (perinatal supplementation: LR‐PS) and *L*. *johnsonii* (LJ‐PS) cocktails of three strains or media only (control: C) were supplemented to dams according to the schematic in Figure S2. Although there was no difference between the weights at any time post‐DSS when comparing any of the groups to each other as a whole (i.e., LR‐PS vs. LJ‐PS vs. C), there was a significant difference in weight loss at day 6 post‐DSS when comparing LR‐PS and C female pups (Figure 4). Therefore, we analyzed microbiome responses to peri‐, and early postnatal supplementation of *Lactobacilli* in the female offspring. There was a progressive microbiome separation by beta diversity with aging (Figure 5A). At P21 (weaning), C separated the most from both LR‐PS and LJ‐PS (*p* = 0.056). By P30, microbiome separation between the groups became significant (*p* = 0.017), which progressed by P70 (pre‐DSS, *p* = 0.001) with LR‐PS separating the most.

The top 10 genera of microbial differences between LR‐PS, LJ‐PS, and C females (based on *p*‐value) were then analyzed at P21, P30, and P70 (Figure 5B). P21 had two genera (*Muribaculum* (*p* = 0.0038) and *Ruminiclostridium* (*p* = 0.0314)) that significantly differed among the groups. P30 had three genera (*Lachnoclostridium* (*p* = 0.0087), *Muribaculum* (*p* = 0.0131), and *Tyzzerella* (*p* = 0.0491) that separated significantly. At P70, eight genera separated significantly between the female groups: *Lachnoclostridium* (*p* = 0.0038; LR‐PS decreased vs. C; 0.01% vs. 0.1%), *Akkermansia* (*p* = 0.0097; LR‐PS decreased vs. C; 13.52% vs. 26.95%), *Lactobacillus* (*p* = 0.0102; LR‐PS increased vs. C; 4.08% vs. 0.82%), *Muribaculum* (*p* = 0.0120; LR‐PS increased vs. C; 2.45% vs. 0%), *Parasutterella* (*p* = 0.0027; LR‐PS decreased vs. C; 0.12% vs. 0.3%), *Ruminiclostridium* (*p* = 0.0190; LR‐PS decreased vs. C; 0.06% vs. 0.25%), *Bacteroides* (*p* = 0.0233; LR‐PS decreased vs. C; 1.13% vs. 2.35%), *Bifidobacterium* (*p* = 0.0330; LR‐PS increased vs. C; 3.59% vs. 1.43%).

Interestingly, only three genera, *Akkermansia*, *Bacteroides*, and *Ruminiclostridium*, differentiated female LR‐PS from both female C and LJ‐PS groups (Figure S5A). *Akkermansia* stood out the most by being decreased in LR‐PS at P70 when compared directly to both C (*p* = 0.0087; 13.52% vs. 26.95%) and LJ‐PS (*p* = 0.0152; 13.52% vs. 21.98%) due to its relatively high abundance. *Bacteroides* was also decreased in LR‐PS at P70 when compared directly to both C (*p* = 0.0152; 1.13% vs. 2.35%) and LJ‐PS (*p* = 0.0260; 1.13% vs. 2.12%). Finally, *Ruminiclostridium* was decreased in LR‐PS at P70 when compared directly to C (*p* = 0.0301; 0.06% vs. 0.25%) and LJ‐PS (*p* = 0.0129; 0.06% vs. 0.24%).

Although the male LR‐PS group was not protected by the maternal *L*. *reuteri* cocktail supplementation, we examined how their microbiomes were affected at P70 (pre‐DSS) compared to control. Male LR‐PS separated out from control by beta‐diversity (Figure S6A). There were less significant genus level differences in male LR‐PS compared to control, however, than in females (Figure S6B). Notably, pre‐DSS *Lactobacillus* was significantly increased in female LR‐PS (4.08% abundance in LR‐PS vs 0.82% in C, *p* = 0.0260 with direct comparison) while there was no difference in the abundance of this genus between male LR‐PS offspring and controls (2.79% abundance in LR‐PS vs. 2.73% in C, *p* = 1.0 with direct comparison). Similarly, *Akkermansia* was not significantly decreased in male LR‐PS compared to controls (Figure S5B; 17.71% abundance in LR‐PS vs. 24.29% in C, *p* = 0.3429 with direct comparison), while the genus was separated in females (Figure S5A; 13.52% abundance in LR‐PS vs. 26.95% in C, *p* = 0.0087 with direct comparison). *Lachnoclostridium* (0.01% abundance in LR‐PS vs. 0.1% in C, *p* = 0.0059 with direct comparison) and *Bacteroides* (1.13% abundance in LR‐PS vs. 2.35% in C, *p* = 0.0152 with direct comparison) were the other two genera that significant separated in females, but not in males (*Lachnoclostridium*: LR‐PS = 0.03%, C = 0.07%, *p* = 0.1720; *Bacteroides*: LR‐PS = 0.76%, C = 2.74%, *p* = 0.0571). Of note, *Alistipes* was significantly decreased in male LR‐PS compared to male C (1.14% vs. 3.22%, *p* = 0.0286) while this separation was not found when comparing female LR‐PS to female C (1.62% abundance in LR‐PS vs. 2.24% in C, *p* = 0.2403 with direct comparison).

*Akkermansia* and *Bacteroides* were both decreased in LR‐PS females (in association with DSS resistance [i.e., protection against colitis]) as opposed to C and LJ‐PS females, and also LR‐PS males (ineffective *Lactobacillus*) (Figure 5). Therefore, these two genera significantly differentiated the perinatally supplemented groups in respect to early adulthood protection against DSS colitis secondary to maternal *L*. *reuteri* supplementation.

## 4 DISCUSSION

### 4.1 Overall findings

This work contains the first preclinical experimental approach to a developmental probiotic by uniquely employing stochastic‐microbiome‐variation directed identification in order to protect against IBD.

### 4.2 Large vendor versus small vivarium

Our mouse model‐based discovery‐validation cohort design allowed us to make several important observations. In the discovery cohort (when mice were sourced from a large vendor), there were obvious microbiome differences prior to exposure of DSS between groups classified as DSS‐R and DSS‐S, notably, increased *Lactobacillus*, likely driven by random, unpredictable differentiation in early life. In the validation cohort, when mice were reared in the same room of our small vivarium, stochastic microbiome variation influencing DSS susceptibility was significantly reduced, importantly lacking *Faecalibaculum*. *Faecalibaculum* has been rarely associated with DSS sensitivity in certain mouse strains.^33^ These findings corroborate prior observations on small vivarium‐induced relative DSS resistance in mice, compared to large vendor bred animals^27^ and implicate *Faecalibaculum* to have participated in the vendor‐originated increase in colitis severity variation of our work. Our results also showed that the convergence of gut microbiomes in a smaller vivarium compared to a large vendor is associated with a reduction in random murine DSS sensitivity. These observations support the prediction that stochastic microbiome variation in mammals is sensitive to environmental influences. Namely, larger environmental variation‐related microbiome divergence is inherently present in large breeding facilities, compared to a single room of a small vivarium. Large vendor‐based microbiome variation in mice may actually be more translatable to humans living in “microbially less tight” communities than coprophagic experimental mice (which share identical diet and environment (i.e., in the same room of a small vivarium)).

### 4.3 Importance of *Lactobacilli*

*Lactobacillus* was a genus in our discovery cohort that significantly associated with protection against DSS colitis (Figure 2B, DSS‐R group). We only uncovered trends for increased DSS‐R *Lactobacillus* abundance in our validation cohort, both at P21 (*p* = 0.13) and P70 (*p* = 0.20) (Figure 3). We took into consideration, however, the existing literature on *Lactobacillus*, and that our validation study cohort was likely underpowered to detect significant microbiome variation toward colitis protection in the setting of the same room in our small vivarium. Therefore, as a proof of concept, we decided to further examine *Lactobacillus* as a genus, which could furnish a developmentally protective probiotic against IBD in our DSS model system.

A study by Je et al.^34^ found that a cocktail comprised of *Lactobacillus johnsonii* IDCC9203, *Lactobacillus plantarum* IDCC3501, and *Bifidobacterium animalis* subspecies *lactis* IDCC4301 was able to reduce symptoms of DSS‐induced colitis in mice. This study also found that the administration of this probiotic cocktail affected these symptoms in a dose‐dependent manner, and also the reduced expression of pro‐inflammatory cytokines such as tumor necrosis factor‐α, interleukin (IL)‐1β, and IL‐6^34^. Kim et al.^35^ found that *Lactobacillus acidophilus* reduced the severity of DSS‐induced colitis and increased the lifespan of the mice by decreasing endoplasmic reticulum stress. Current literature on probiotics affecting human IBD, however, is limited and needs further research.^36^ A review by Coquiero et al.^37^ found that probiotic supplementation, especially with *Lactobacillus*, presented a possible therapeutic option for induction, maintaining remission, or decreasing symptoms in UC. No human or animal studies, however, have examined perinatally delivered probiotics as a preventative against the development of IBD or experimental murine colitis later in life.

### 4.4 Perinatal administration of a probiotic

A study by Atarashi et al.^38^ observed that oral inoculation of *Clostridium* in infant mice led to an increase in regulatory T cells and colitis resistance, but they started supplementation two weeks after birth, and did not identify any changes with *Lactobacillus* supplementation. Liu et al.^39^ observed that early supplementation (from P8 to P20) of *L*. *reuteri* DSM 17938 in mice promoted microbial diversity, increased immune tolerance through a higher proportion of Foxp3+ regulatory T cells in the intestine, and stimulated plasma metabolites that enhance tolerance to inflammatory stimuli. In humans, a randomized controlled trial by Schmidt et al.^40^ found that supplementation of a probiotic containing *Lactobacillus rhamnosus* and *Bifidobacterium animalis* subsp *lactis* during late infancy decreased the incidence of eczema compared to a control group. Canova et al.^41^ conducted a prospective study that discovered early life exposure to antibiotics increased the risk of childhood‐onset IBD, supporting the idea that early microbial influences may modulate the risk of subsequent IBD development. Therefore, based on our results in our discovery and validation cohorts, we decided to test two probiotic cocktails, containing three *Lactobacillus* strains each, as a perinatal/early postnatal intervention to decrease subsequent DSS colitis susceptibility in young adult mice. This approach allowed us to determine whether a transiently delivered, developmental probiotic could induce protection against mammalian colitis susceptibility.

### 4.5 Sexual dimorphism in probiotic response

Maternal *L*. *reuteri* cocktail supplementation successfully transferred DSS resistance to young adult LR‐PS female offspring (Figure 4). Intriguingly, we observed a progressively increasing microbiome separation from P21 to P70 (pre‐DSS) in the pups following maternal *L*. *reuteri* administration (Figure 5A). Male LR‐PS microbiomes also separated from control, but to a lesser degree than females (Figure S6). We speculate that estradiol driven^42^ or other sex‐specific differences in gut mucosal biology^43^ may be the reason for this gender‐based separation in mice. Sexual dimorphism has been previously observed to factor in the effectiveness of probiotic use in mice affecting intestinal inflammation and bone density.^44^

### 4.6 Notable microbes

*Lactobacillus*, *Muribaculum*, and *Bifidobacterium* were significantly associated with DSS protection in the female LR‐PS group while *Lachnoclostridium*, *Akkermansia*, *Parasutterella*, *Ruminiclostridium*, and *Bacteroides* were associated with DSS susceptibility (i.e., increased abundance in female C vs. female LR‐PS). When comparing the female LR‐PS (DSS protected group) to female LJ‐PS and male LR‐PS groups (i.e., maternally *Lactobacillus* supplemented, but not protected against DSS), the relative decrease of *Akkermansia* and *Bacteroides* stood out most. In humans, decreased amounts of *Akkermansia* have been associated with UC at diagnosis (i.e. during the state of inflammation^45^). *Akkermansia* and *Bacteroide*s harbor extensively glycosyl hydrolases, which allow them to systematically degrade the protective mucus layer.^46^ Mucolytic bacteria have been shown to be increased in the mucosa of IBD patients^47^, ^48^, ^49^ and the degradation of the mucus layer has been hypothesized to contribute to the inflammatory milieu. It has been speculated that the degradation of mucus by mucolytic microbes in IBD patients provides a nutrient source for non‐mucolytic mucosa‐associated bacteria. In mice, mucin‐degrading microbes have been shown to promote the expansion of enteric pathogens and viruses,^46^, ^50^, ^51^ and thus may inadvertently promote inflammation. Therefore, a decreased amount of *Akkermansia* may be protective against IBD by allowing for a more pronounced and efficient mucus barrier layer at the colonic mucosa. Recent work on *Bacteroides*‐supplementation found *Akkermansia* to be associated with therapeutic failure in respect to DSS protection, supporting this idea.^29^ Conversely, in a SH2‐domain‐containing inositol 5′‐phosphatase (SHIP)‐deficient mouse model (a model of Crohn's disease), *Bacteroides* species were enriched prior to inflammation,^52^ which is consistent with the decreased abundance of *Bacteroides* in our DSS resistant LR‐PS female offspring of our study. Additionally, in a study by Wu et al.^53^ investigating the impact of triptolide on DSS‐induced colitis, decreased amount of *Bacteroides* was associated with anti‐inflammatory effects, including significantly increased microbial diversity. Based on our data, we speculate that supplementation of *L*. *reuteri* promoted a more anti‐inflammatory pro‐host microbiome, which in our mouse model consisted of decreased *Akkermansia* and *Bacteroides*.

Another key finding was that our *L*. *reuteri* cocktail increased the levels of *Bifidobacterium*. Similar to our study, oral administration of *Bifidobacterium breve* M1 and M2 was found to ameliorate DSS‐induced colitis while reducing the abundance of *Bacteroides* simultaneously.^54^ In mice, *Bifidobacteria* beneficially modulate the protective mucus layer and *B*. *lactis*, *B*. *bifidum*, and B. *longum* have been shown to attenuate colitis and inflammation.^55^, ^56^, ^57^, ^58^, ^59^, ^60^ The importance of *Bifidobacteria* is also highlighted by the fact that *Bifidobacteria* are depleted in IBD.^61^, ^62^ We speculate that our *Lactobacilli* cocktail may be working in synergy with *Bifidobacteria* to inhibit colitis. Therefore, in our study, maternal supplementation of an *L*. *reuteri* cocktail most likely transferred colitis protection by modulating the evolution of the gut microbiome as a whole toward a less colitis prone state.

### 4.7 Study limitations

We acknowledge several limitations to our work, and highlight the proof‐of‐concept, paradigm‐shifting intention of it. Since we were aiming to study the difference of the two extremes on the distribution curve for DSS susceptibility (Figure S1), an ideal scenario would have been to include about 1000 mice (based on a power calculation) in our experiment and compare the most and least protected 10–15 animals’ microbiomes in the DSS‐R and DSS‐S groups from that large cohort. Due to the obvious cost and feasibility constraints, our experiment was limited to a much smaller sample size. A discovery‐validation cohort approach was utilized in an attempt to decrease the small group size limitations on stochastic microbiome variation.

Another constraint was in the depth of taxonomic identification in this study using *16S rRNA* sequencing. Therefore, we did not confirm specifically the transfer of the experimental *Lactobacillus* strains into the dams and pups, but rather focused on the secondary functional (i.e. DSS resistance) and genus level microbiome changes that our intervention induced. This approach was used due to cost‐benefit considerations for our specific experiment since shotgun sequencing (or other means of detailed sequencing) is significantly more expensive for an arguably limited payoff. Namely, our goal was to introduce a novel conceptual approach toward the identification of a developmental probiotic against human IBD in an imperfect animal model, where specific microbiome associations may bare limited translational value. We also recognize that our probiotic identification was done in males, yet the beneficial effect from those occurred in females only. This finding repeatedly underscores the unpredictable nature of microbiome responses, whereby such approaches as ours may only provide taxonomic targets for manipulation, but not necessarily the means by which those targets can be achieved (i.e. increased *Lactobacilli* at P70 in males was not achieved by perinatal [maternal] supplementation of those, for example).

We only examined a single, but objective outcome measure of DSS colitis, namely weight loss. However, we do not find this a limitation. We intentionally aimed to further a paradigm shift toward the mouse DSS colitis model. We have recently underscored that in more than 3,000 peer‐reviewed publications that used the DSS model, weight loss has been the most economic, most consistent, and objective measurement of colitis severity.^28^ Consequently, we wished to demonstrate the importance of the judicious use or research resources, especially during the COVID‐19 pandemic, and show that weight loss is a sufficient single marker of DSS colitis at current state of art.

In spite of the limitations, we are confident that our findings in addition to those by Schmidt et al.^40^ (see earlier, where infantile *Lactobacillus rhamnosus* and *Bifidobacterium animalis subsp lactis* protected against eczema in a clinical trial) position *Lactobacilli* as outstanding candidates for translational studies in humans as a developmental (perinatally delivered) probiotic against IBD. Importantly, genetic predisposition to eczema and IBD is shared,^63^ and a bidirectional clinical association between IBD and eczema (i.e., atopic dermatitis [AD]) appears to exist.^64^ Furthermore, some similarities between AD‐ and CD‐associated microbiomes have been observed.^65^ In the meantime, only a very large cohort, placebo‐controlled prospective trial with prolonged (i.e., through the first 30 years of life for the participants at minimum) follow‐up could provide answers for perinatal/early postnatal microbial therapeutics to prevent against IBD.

## CONFLICT OF INTEREST

The authors have no conflicting interests to declare.

## AUTHOR CONTRIBUTIONS

Mahesh Krishna performed data collection, data analysis, and drafted the manuscript. Melinda Engevik performed data collection and contributed new reagents. Karen Queliza, Savini Britto, Wenly Ruan, and Hongtao Wang performed data collection. Rajesh Shah performed data analysis. James Versalovic contributed to conceptual design, with reagents, and manuscript review. Richard Kellermayer performed the conceptual design, data analysis and manuscript writing, and obtained funding.

## Supporting information

Fig S1‐S6
