# Supplementary material for: Maternal Lactobacillus reuteri supplementation shifts the intestinal microbiome in mice and provides protection from experimental colitis in female offspring
Source: FASEB Bioadv. 2021 Nov 1;4(2):109–20. doi: 10.1096/fba.2021-00078 (PMC8814561; doi:10.1096/fba.2021-00078)
Supplement: Supplementary file 1 — Fig S1‐S6 [file FBA2-4-109-s001.pptx]

## Slide 1
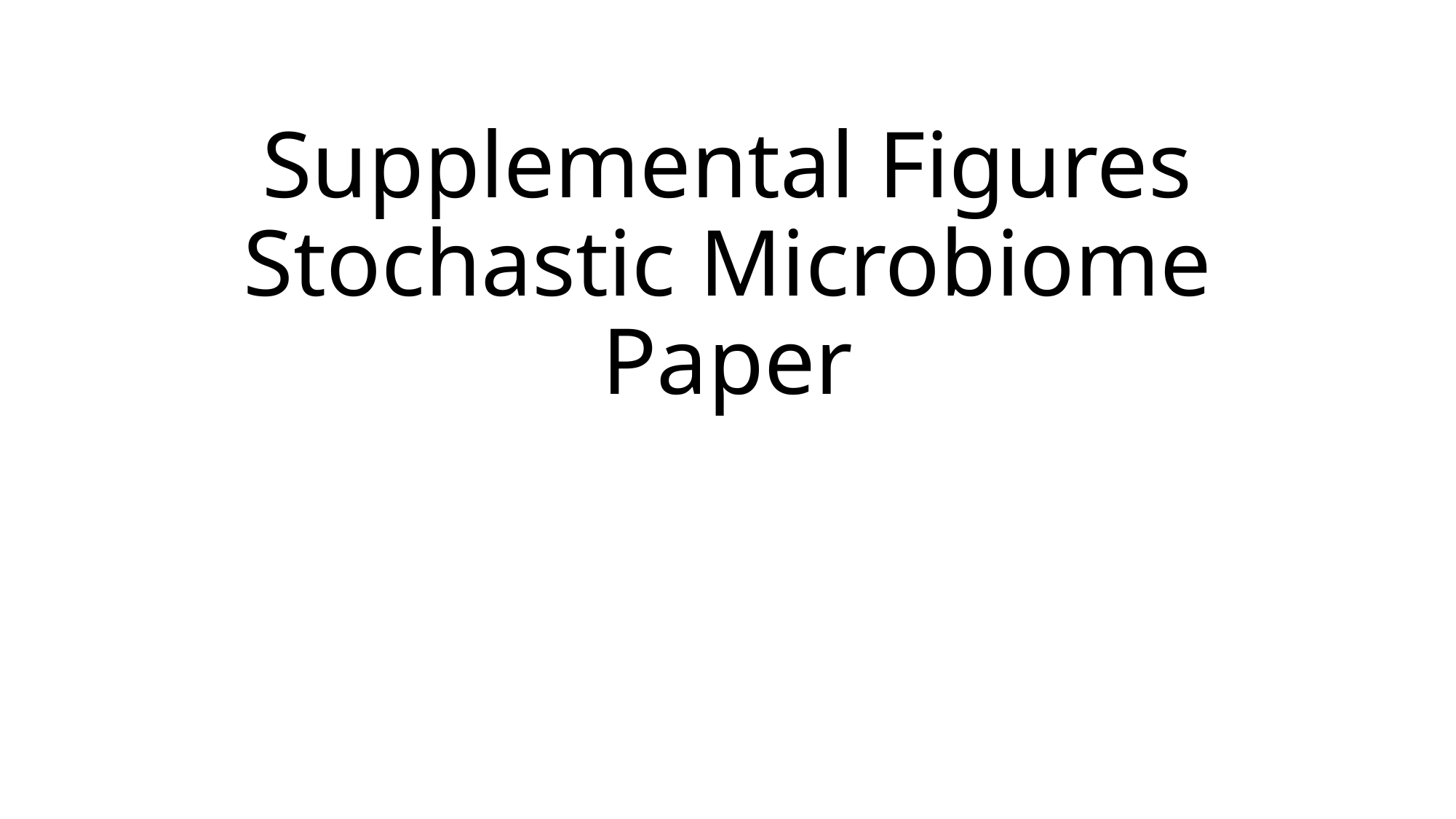

# Supplemental Figures Stochastic Microbiome Paper

## Slide 2
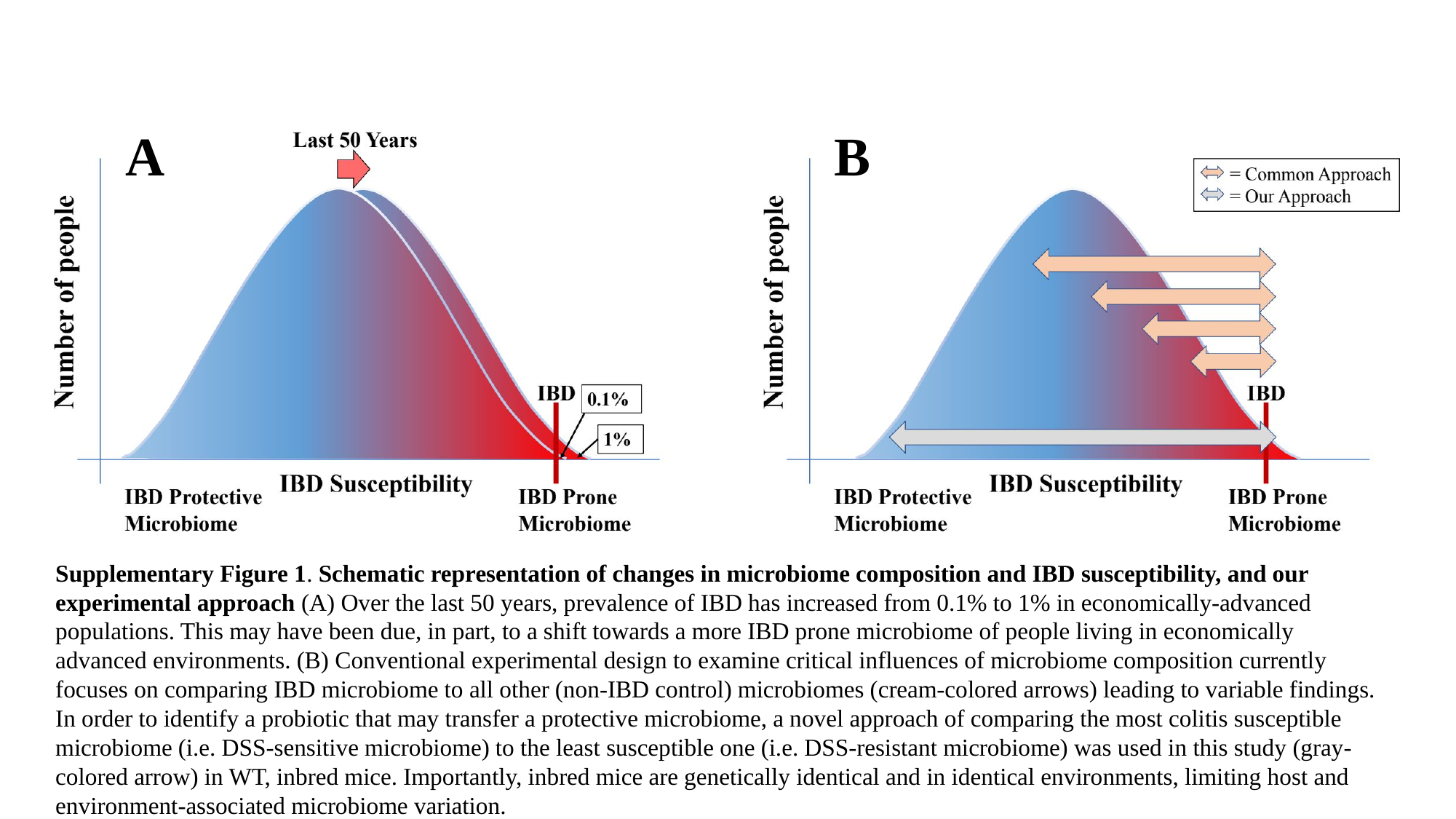

B
A
Supplementary Figure 1. Schematic representation of changes in microbiome composition and IBD susceptibility, and our experimental approach (A) Over the last 50 years, prevalence of IBD has increased from 0.1% to 1% in economically-advanced populations. This may have been due, in part, to a shift towards a more IBD prone microbiome of people living in economically advanced environments. (B) Conventional experimental design to examine critical influences of microbiome composition currently focuses on comparing IBD microbiome to all other (non-IBD control) microbiomes (cream-colored arrows) leading to variable findings. In order to identify a probiotic that may transfer a protective microbiome, a novel approach of comparing the most colitis susceptible microbiome (i.e. DSS-sensitive microbiome) to the least susceptible one (i.e. DSS-resistant microbiome) was used in this study (gray-colored arrow) in WT, inbred mice. Importantly, inbred mice are genetically identical and in identical environments, limiting host and environment-associated microbiome variation.

## Slide 3
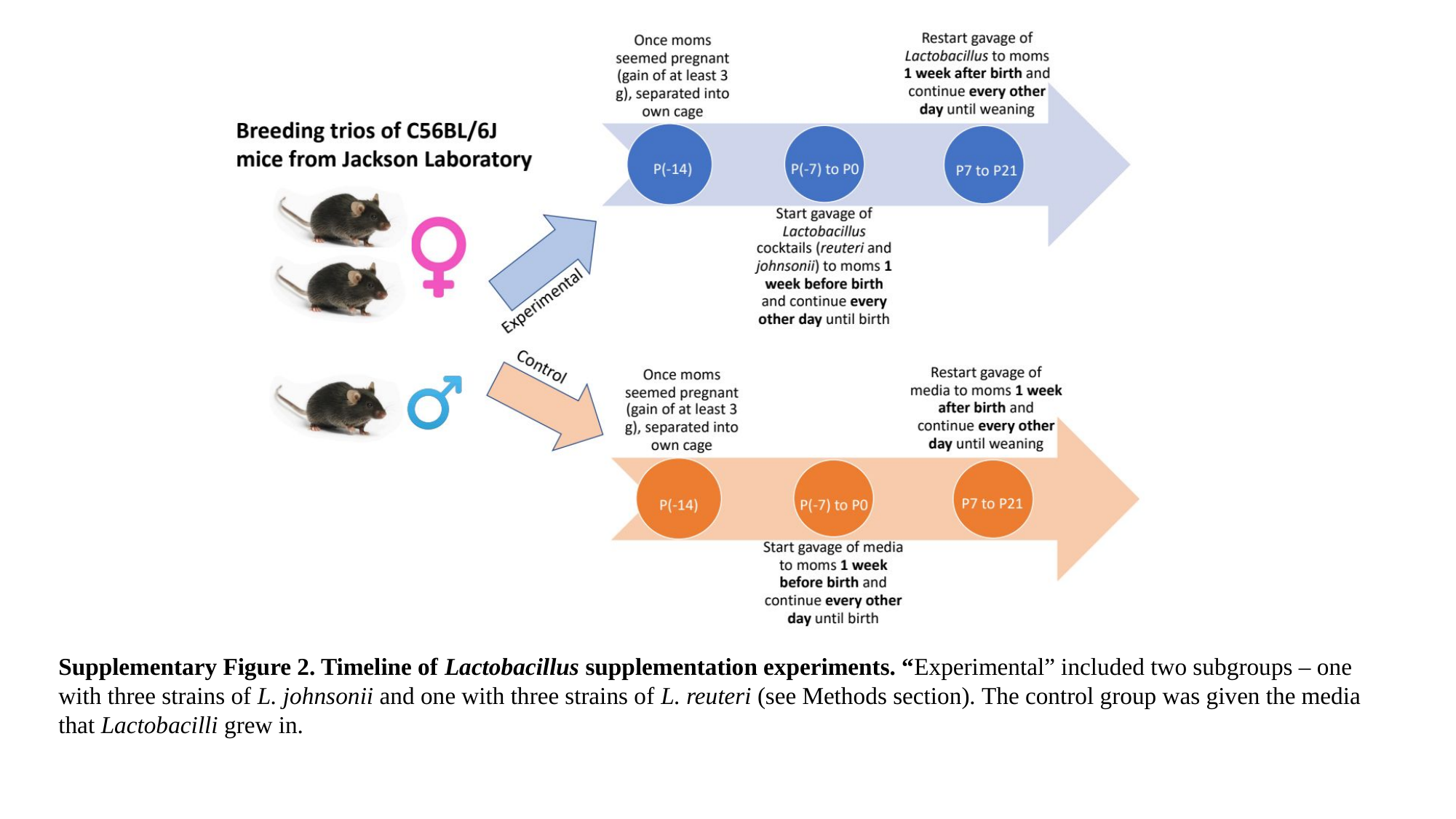

Supplementary Figure 2. Timeline of Lactobacillus supplementation experiments. “Experimental” included two subgroups – one with three strains of L. johnsonii and one with three strains of L. reuteri (see Methods section). The control group was given the media that Lactobacilli grew in.

## Slide 4
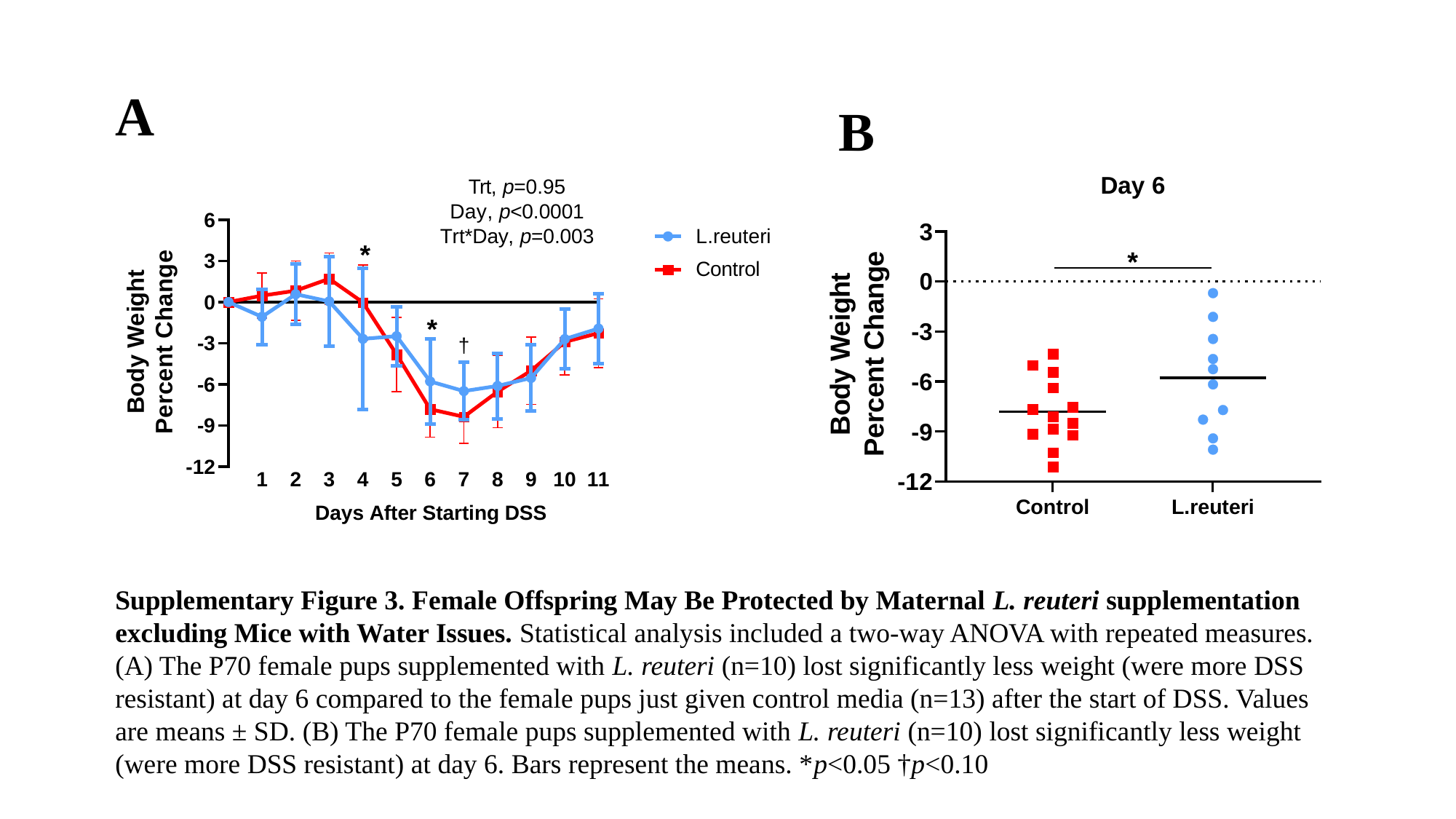

A
B
Supplementary Figure 3. Female Offspring May Be Protected by Maternal L. reuteri supplementation excluding Mice with Water Issues. Statistical analysis included a two-way ANOVA with repeated measures. (A) The P70 female pups supplemented with L. reuteri (n=10) lost significantly less weight (were more DSS resistant) at day 6 compared to the female pups just given control media (n=13) after the start of DSS. Values are means ± SD. (B) The P70 female pups supplemented with L. reuteri (n=10) lost significantly less weight (were more DSS resistant) at day 6. Bars represent the means. *p<0.05 †p<0.10

## Slide 5
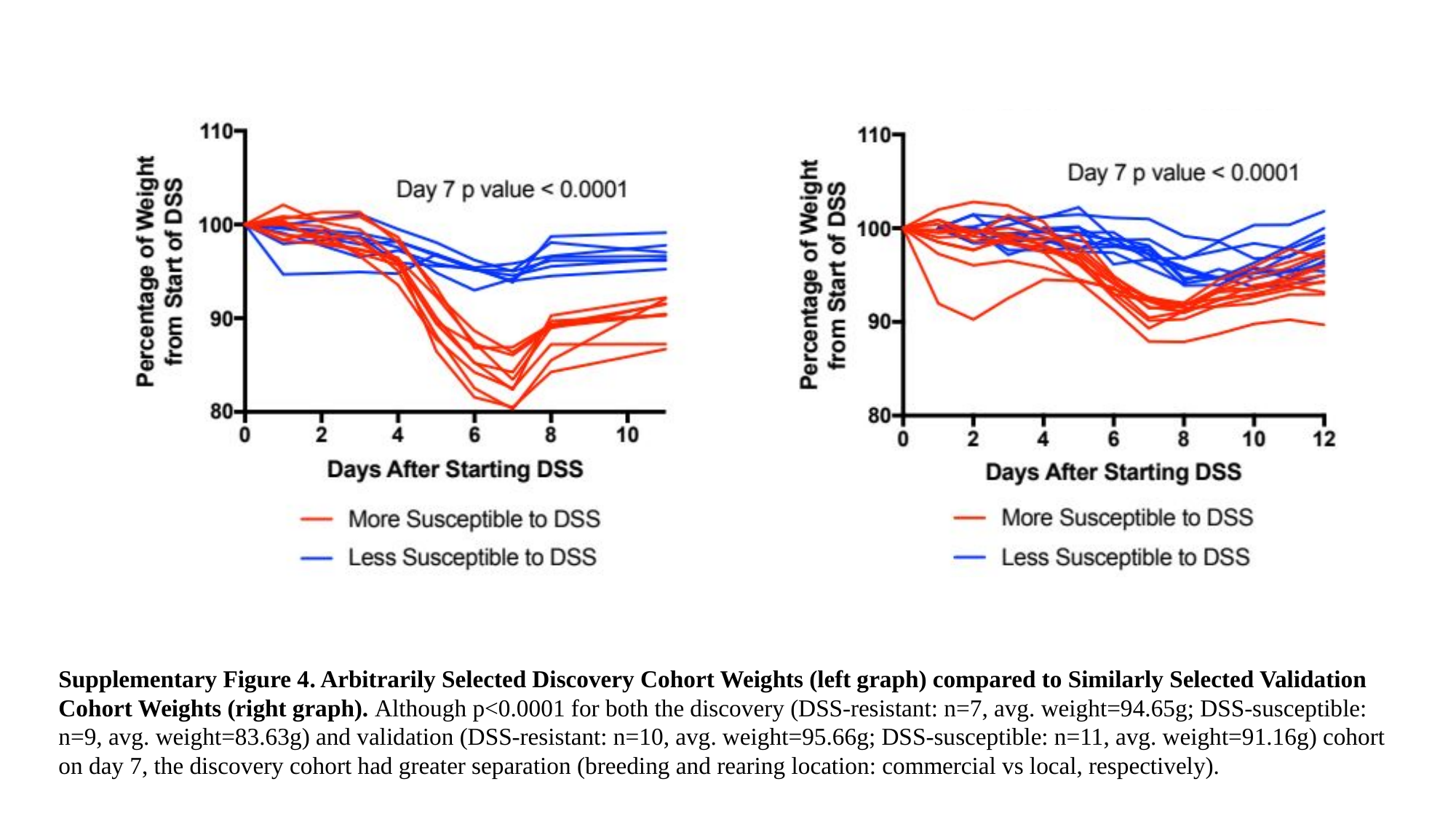

Supplementary Figure 4. Arbitrarily Selected Discovery Cohort Weights (left graph) compared to Similarly Selected Validation Cohort Weights (right graph). Although p<0.0001 for both the discovery (DSS-resistant: n=7, avg. weight=94.65g; DSS-susceptible: n=9, avg. weight=83.63g) and validation (DSS-resistant: n=10, avg. weight=95.66g; DSS-susceptible: n=11, avg. weight=91.16g) cohort on day 7, the discovery cohort had greater separation (breeding and rearing location: commercial vs local, respectively).

## Slide 6
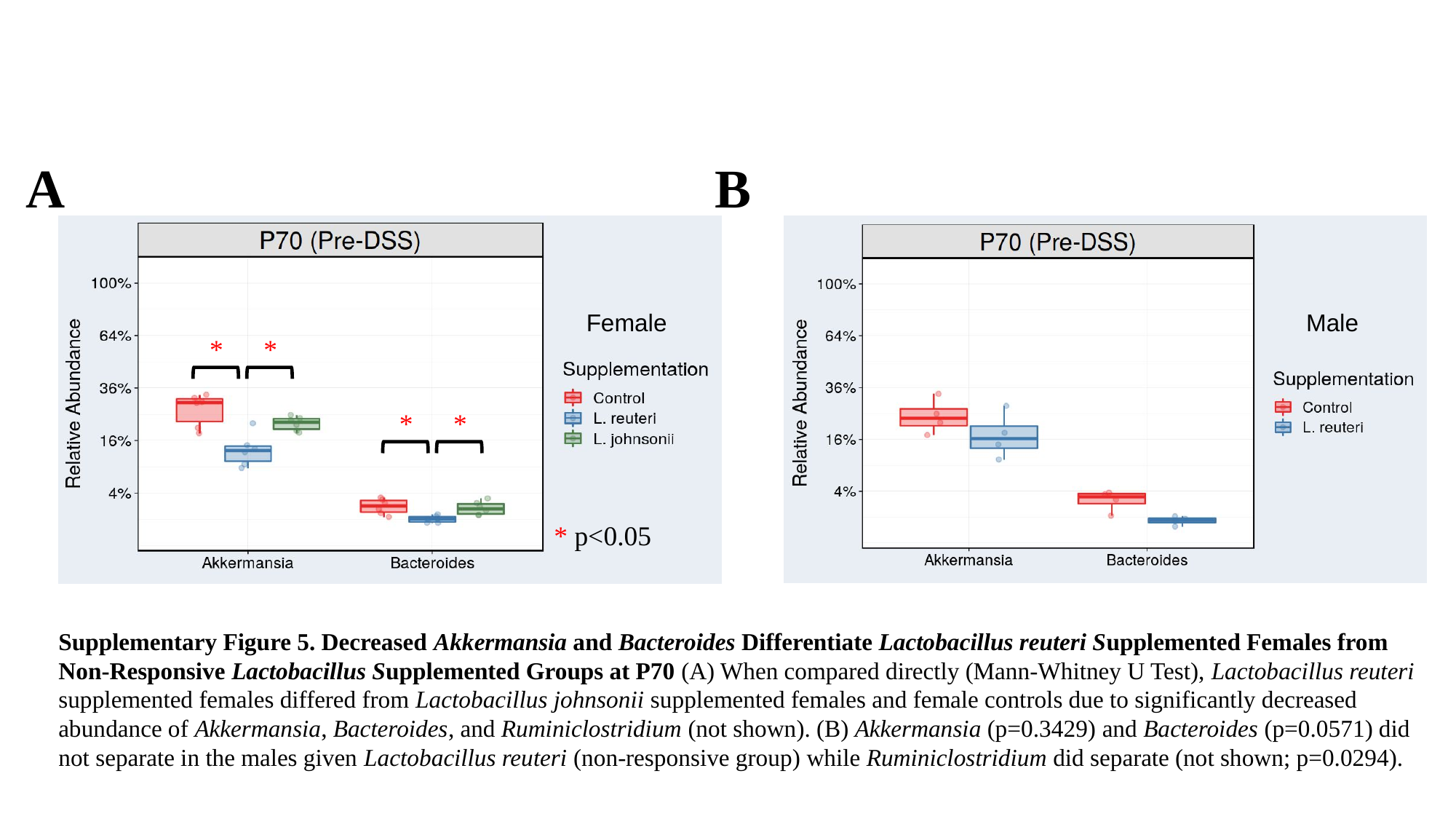

A
B
*
*
*
*
Female
Male
* p<0.05
Supplementary Figure 5. Decreased Akkermansia and Bacteroides Differentiate Lactobacillus reuteri Supplemented Females from Non-Responsive Lactobacillus Supplemented Groups at P70 (A) When compared directly (Mann-Whitney U Test), Lactobacillus reuteri supplemented females differed from Lactobacillus johnsonii supplemented females and female controls due to significantly decreased abundance of Akkermansia, Bacteroides, and Ruminiclostridium (not shown). (B) Akkermansia (p=0.3429) and Bacteroides (p=0.0571) did not separate in the males given Lactobacillus reuteri (non-responsive group) while Ruminiclostridium did separate (not shown; p=0.0294).

## Slide 7
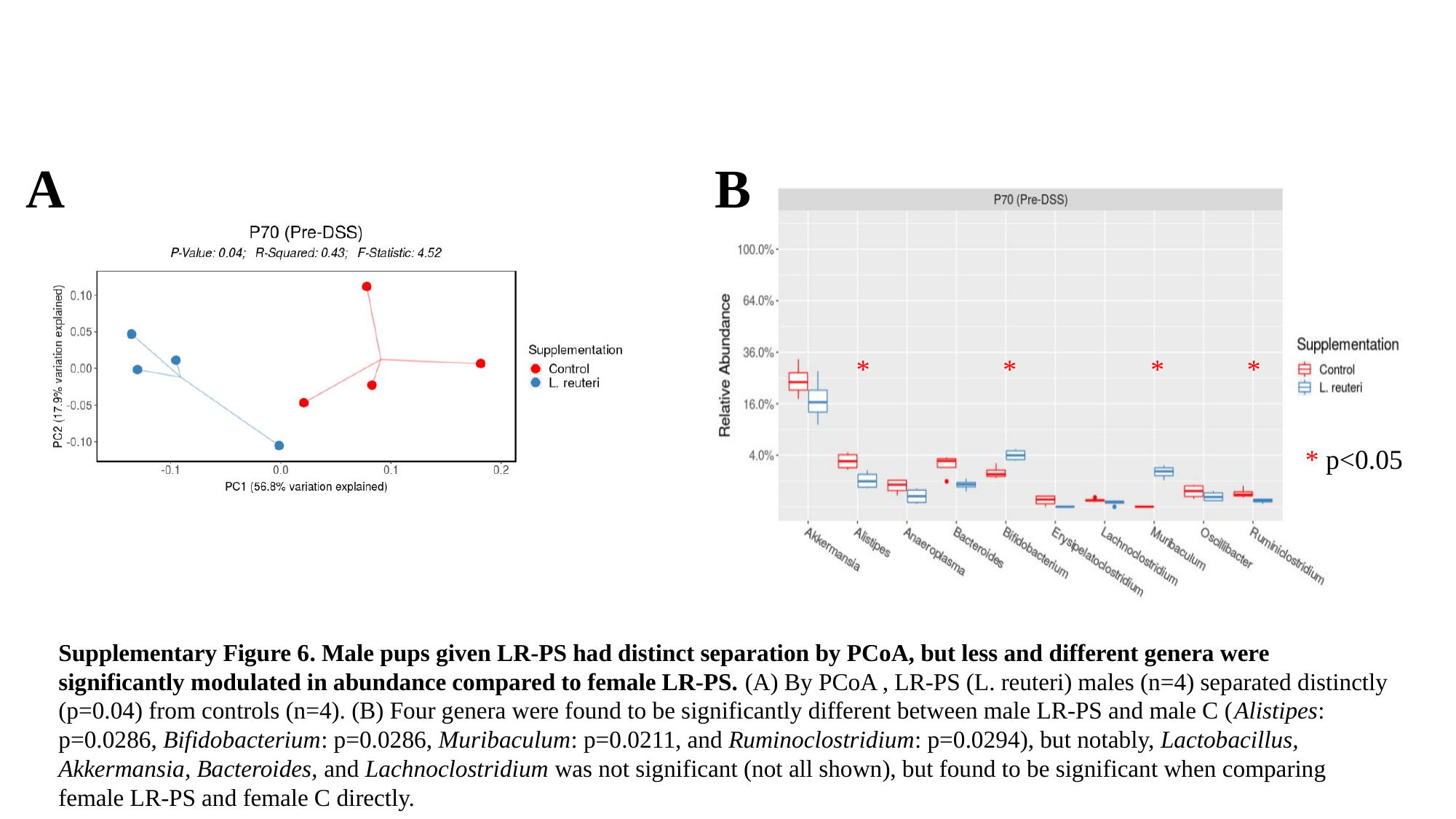

A
B
*
*
*
*
* p<0.05
Supplementary Figure 6. Male pups given LR-PS had distinct separation by PCoA, but less and different genera were significantly modulated in abundance compared to female LR-PS. (A) By PCoA , LR-PS (L. reuteri) males (n=4) separated distinctly (p=0.04) from controls (n=4). (B) Four genera were found to be significantly different between male LR-PS and male C (Alistipes: p=0.0286, Bifidobacterium: p=0.0286, Muribaculum: p=0.0211, and Ruminoclostridium: p=0.0294), but notably, Lactobacillus, Akkermansia, Bacteroides, and Lachnoclostridium was not significant (not all shown), but found to be significant when comparing female LR-PS and female C directly.
